# The PSC scientific community resource: an asset for multi-omics interrogation of primary sclerosing cholangitis

**DOI:** 10.1186/s12876-021-01930-2

**Published:** 2021-09-25

**Authors:** Ahmad Hassan Ali, Brian D. Juran, Erik M. Schlicht, Jackie K. Bianchi, Bryan M. McCauley, Elizabeth J. Atkinson, Konstantinos N. Lazaridis

**Affiliations:** 1 Division of Gastroenterology and Hepatology, Mayo Clinic, 200 First Street SW, Rochester, MN 55905 USA; 2 Division of Biomedical Statistics and Informatics, Mayo Clinic, Rochester, MN USA

**Keywords:** Cholangitis, Biobank, Pathogenesis, Exposome, Metabolome, Methylome, Microbiome, Cirrhosis

## Abstract

**Background:**

Primary sclerosing cholangitis (PSC) is a rare, chronic cholestatic liver disease that often progresses to end-stage liver disease and/or the development of hepatobiliary neoplasia. Lack of prognostic tools and treatment options for PSC is driven in part by our poor understanding of its pathogenesis, which is thought to be complex, the interaction of genetic variants, environmental influences and biological response throughout the course of disease. The PSC Scientific Community Resource (PSC-SCR) seeks to overcome previous shortcomings by facilitating novel research in PSC with the ultimate goals of individualizing patient care and improving patient outcomes.

**Methods:**

PSC patients who receive their health care at Mayo Clinic or a collaborating site are identified by chart review and invited in person or by mail to participate. Non-Mayo patients are offered enrollment if they provide sufficient access to their medical records to evaluate inclusion/exclusion criteria. Controls without liver disease are identified with assistance of the Mayo Clinic Biobank. Participant consent is obtained at the beginning of the recruitment process by mail-in, electronic or face-to-face protocols. Clinical data is extracted from the medical record by qualified physicians and entered in a custom designed database. Participants fill out a custom-designed, comprehensive questionnaire, which collects scientifically relevant demographic and clinical information. Biospecimens are collected using mail-in kits thar are returned via overnight carrier service and processed by the biospecimen accessioning and processing facility at Mayo Clinic, which coordinates sample transfers and provides required sample preparation services. The resource is currently being utilized to perform omics-scale projects investigating the exposome, metabolome, methylome, immunome and microbiome in PSC. Datasets and residual biospecimens will be shared with researchers proposing scientifically sound PSC-focused research with approval of the appropriate review boards.

**Discussion:**

Patient-based studies leveraging the latest technologies for targeted and wide-scale interrogation of multiple omics layers offer promise to accelerate PSC research through discovery of unappreciated aspects of disease pathogenesis. However, the rarity of PSC severely limits such studies. Here we describe our effort to overcome this limitation, the PSC-SCR, a repository of patient biospecimens coupled with clinical and omics data for use by the broader PSC research community.

**Supplementary Information:**

The online version contains supplementary material available at 10.1186/s12876-021-01930-2.

## Background

Primary sclerosing cholangitis (PSC) is a fibroinflammatory disease of the bile ducts resulting in chronic cholestasis often progressing to end-stage liver disease and/or the development of hepatobiliary neoplasia [1]. No effective medical treatments to slow disease progression exist for PSC outside of liver transplantation, which is not always curative as disease recurs in about 25% of transplanted patients [2]. PSC is a rare disease, with reported incidence ranging from 0.07 to 1.3 per 100,000 people and reported prevalence ranging from 0.2 to 16.2 per 100,000 people [3]. Clinical presentation of PSC is heterogeneous, it can affect both small and large intrahepatic and/or extrahepatic bile ducts and is often, but not always, associated with concurrent inflammatory bowel disease, which occurs in ~ 70% of patients [4]. PSC has a substantial socioeconomic burden. Many patients receive costly procedure-based surveillance and data have shown that PSC is the fifth leading indication for liver transplantation in the United States [5] and is the main indication for liver transplantation in some Nordic countries [6]. Rate of disease progression in PSC is highly variable and prediction of when, or in who, the feared outcomes end-stage liver disease and hepatobiliary cancer will develop remains difficult [4].

The risk of developing hepatobiliary cancer has been estimated to be significantly higher in PSC patients compared to the general population [7]. Cholangiocarcinoma (CCA), a primary bile duct cancer, is the most common and feared cancer in PSC [8], with reported lifetime risk ranging between 6 and 30% [9]. CCA is one of the most aggressive tumors of the biliary tract/liver, with a reported median survival in all anatomical types without surgery ranging between 5 and 12 months [10]. Unfortunately, diagnosis of early-stage CCA is often extremely difficult due to the lack of reliable diagnostic biomarkers, and thus, many patients with CCA are not eligible for curative surgery due to advance disease stage at diagnosis [10].

Lack of prognostic tools and treatment options for PSC is driven in part by our poor understanding of its pathogenesis, which is thought to be complex, the interaction of homeostatic perturbations driven by genetic variants, environmental influences and biological response throughout the course of disease [11]. Several processes have been proposed to play a role in PSC pathogenesis such as bile acid toxicity [12, 13], diminished biliary bicarbonate umbrella [14], aberrant lymphocyte homing [15, 16], leaky gut [17] and intestinal dysbiosis [18]. Genome-wide association studies have offered additional insights into likely PSC risk factors and highlight the role of immunity in PSC development [19, 20]. However, due to multiple hinderances, previous research efforts in PSC have been largely limited in scope and translation. Patient-based longitudinal studies of PSC are severely restricted by low disease incidence, lack of informative biomarkers in early stages of disease, and relative inaccessibility of the biliary tract [1]. Moreover, while some of the current animal models do demonstrate individual features of PSC, none display the constellation of characteristics commonly observed in patients [21]. Thus, use of these models is largely limited to focused studies, the findings of which may not be truly reflective of human disease. Finally, while the environment is thought to have a significant influence on PSC pathogenesis, studies have been limited to recall-based questionnaires [22], which lack precision to identify disease associations.

The PSC Scientific Community Resource (PSC-SCR) seeks to overcome previous shortcomings by facilitating novel research in PSC through creation of a resource of biospecimens and -omics scale data from patients with PSC and relevant controls. This resource will be made available to the broader community of PSC-interested researchers, bringing together investigators with a wide range of expertise to accelerate the pace of PSC research. The ultimate goals are to individualizing care of patients with PSC care and improving their outcomes.

The PSC-SCR is comprised of biospecimens and clinical data collected under two biobank-focused IRB protocols as well as research data generated using specimens obtained from the resource. The first protocol (Mayo Clinic IRB #670-02), which is no longer enrolling subjects, was primarily designed to collect blood specimens to facilitate robust studies of genetic and environmental contributors to PSC using DNA and questionnaire-based interrogations. However, additional sample types including plasma, serum and peripheral blood mononuclear cells (PBMC) were collected and are available for use in PSC research. The second protocol (Mayo Clinic IRB #16-005892), which is actively enrolling subjects, has the expanded scope to collect specimens to facilitate a wide range of -omics scale studies. This protocol collects additional sample types from blood, including RNA and circulating cell-free DNA, as well as stool and urine specimens. Both protocols collect(ed) specimens and data from PSC patients and controls and the current protocol is open to individuals enrolled in the earlier one. Where necessary to discriminate between the two protocols in the remainder of the manuscript we will refer to them as the “current” (Mayo Clinic IRB #16-005892) or “previous” (Mayo Clinic IRB #670-02) protocols.

## Methods

### Enrollment of patients and controls

#### Patient recruitment

PSC patients who receive their health care at one of the three primary Mayo Clinic sites (Minnesota, Florida, and Arizona) or in the broader Mayo Clinic Health System are identified by chart review and invited in person or by mail to participate in our studies. Non-Mayo patients are offered enrollment if they provide sufficient access to their medical records to evaluate inclusion/exclusion criteria. We also engage in collaboration with clinicians and researchers from other non-Mayo medical centers to increase patient enrollment while balancing the need to obtain sufficient medical data to perform informed studies with privacy concerns of patients and the collaborating centers. Our previous protocol included seven such collaborating medical centers: Indiana University, University of Toronto, Virginia University Medical Center, Mount Sinai Hospital, Virginia Commonwealth University, Johns Hopkins University and University of Pittsburgh. Our current protocol is engaged in two such collaborations: Indiana University and University of Miami. Interested clinicians/researchers are encouraged to contact us regarding referring their patients to our study or potential formal collaboration.

#### Control subjects

Individuals without liver disease who receive their health care at one of the three primary Mayo Clinic sites (Minnesota, Florida, and Arizona) or in the broader Mayo Clinic Health System are identified by chart review and invited in-person or by mail to participate in our studies. Our previous protocol focused on recruitment of control patients receiving care through the outpatient clinics of the Division of Preventive Medicine and the Division of General Internal Medicine. Our current protocol identifies potential controls in collaboration with the Mayo Clinic Biobank [23], and seeks to roughly match the composition of recruited patients based on age, sex and region of residence. Of note, this control population is shared with our similarly sized biobank of PBC patients, which is not described in this manuscript.

#### Enrollment criteria

In our current protocol, patients and controls of any sex and racial or ethnic background between the ages of 18 and 85 at study entry are eligible for enrollment. Whereas our previous protocol also allowed for children between the ages of 5 and 18 to enroll using a modified consent process and sample collection kit. Pregnant women and women of childbearing age can enroll as the protocol does not pose a threat to pregnancy. All PSC patients must meet the following established diagnostic criteria for PSC to be enrolled in the study: (a) biochemical evidence of chronic cholestasis (≥ 6 months, duration); (b) cholangiographic evidence of multifocal strictures and segmental dilatations in the bile ducts and/or histological features consistent with PSC; and (c) exclusion of secondary causes of sclerosing cholangitis [24]. The resource includes patients with variant forms of PSC such as small-duct disease and PSC overlapping with autoimmune hepatitis; as well as patients who have previously received a liver transplant. Patients with other concurrent liver disease (besides for autoimmune hepatitis) or who are unable to provide informed consent are not eligible for enrollment. Currently, exclusion criteria for controls includes documented history of cholestatic or other chronic liver disease, inflammatory bowel disease, or history of organ transplant.

#### Consent process

Participant consent is obtained at the beginning of the recruitment process by mail-in, electronic or face-to-face protocols. Patients are encouraged to contact the study staff with any questions and are informed that their response will not impact their medical care. Our protocols have been reviewed by the Mayo Clinic IRB and all patients provide informed consent for use of their data and biospecimens for current and future research.

### Chart review

Patient clinical data is extracted from the Mayo Clinic’s electronic and non-electronic medical records by qualified physicians and manually entered into the PSC Study Data Management System (PSC-SDMS), a custom-designed SAS-based electronic data capture web application developed by the Mayo Clinic’s Biomedical Informatics Support Systems section. The PSC-SDMS database is secure and password-protected and patient records are updated at regular intervals.

Clinical data collected include demographic characteristics such as gender, BMI, self-reported race and date of birth; as well as PSC-related features such as date of PSC diagnosis, pattern/type of biliary ductal involvement, histological stage / biopsy results, and IBD status. Data reflecting features related to the development of PSC endpoints such as development of cirrhosis, hepatic decompensation, liver transplantation and development of PSC-related malignancies are also documented. A complete list of clinical data collected from PSC patients and stored in the PSC-SDMS is provided in Table 1. In addition to manually curated data, results of clinical lab assessments, such as liver function tests, are extracted and entered in PSC-SDMS using an automated process.

**Table 1:** Patient data collected in the PSC-SDMS database

Category	Variables
Patient demographics	Date of birth, Sex, Ethnicity, Race, Weight, Height, BMI
Chart review	Date of chart review, Date of last clinical encounter, Inclusion/Exclusion, Vital status (date and cause of death, if applicable)
PSC diagnosis	Date of diagnosis, Type of PSC (small duct, large duct), PSC location (intrahepatic, extrahepatic, both), Symptoms presenting at diagnosis, Overlap with autoimmune hepatitis, Other concurrent liver disease
Liver Biopsy	Yes/no, Dates, Histological stage (Ludwig’s system)
ERCP	Yes/no, Dates, FISH polysomy findings, Cytology findings
MRE	Yes/no, Dates, Liver stiffness findings
Dominant stricture	Yes/no, Date, Location of stricture
Disease complications	Cirrhosis (yes/no, date), Ascites (yes/no, date), Splenomegaly (yes/no, date), Varices (yes/no, date), Variceal hemorrhage (yes/no, date), Hepatic encephalopathy (yes/no, date)
Hepatobiliary cancer	Cholangiocarcinoma (yes/no, date), Hepatocellular carcinoma (yes/no, date), Gallbladder cancer (yes/no, date)
Liver transplantation	Listed for transplant (yes/no, date), Received transplant (yes/no, dates, indications, donor types)
IBD assessment	Yes/no, Type of IBD (UC, CD, indeterminate), Colon biopsy (yes/no, dates, findings), Pouchitis (yes/no, date), Proctocolectomy (yes/no, date), Colon dysplasia (yes/no, date), Colorectal cancer (yes/no, date)
Medications	UDCA (yes/no, dose, dates), Prednisone (yes/no, dates), 5-aminosalicyclic acid (yes/no, dates), Azathioprine (yes/no, dates), Mercaptopurine (yes/no, dates), Biological therapy for IBD (yes/no, dates)
Concurrent Autoimmunity (pulled from EMR)	Autoimmune thyroid disease (yes/no, date, type), Rheumatoid arthritis (yes/no, date), Type 1 diabetes (yes/no, date), Psoriasis (yes/no, date), Celiac disease (yes/no, date), Raynaud’s syndrome (yes/no, date), Scleroderma (yes/no, date), Sjogren’s syndrome (yes/no, date), Systemic lupus erythematosus (yes/no, date), Vitiligo (yes/no, date), other autoimmunity (specify)
Laboratory tests (pulled from EMR)	Alkaline phosphatase, aspartate aminotransferase, alanine aminotransferase, bilirubin (total and direct), albumin, sodium, creatinine, platelets, hemoglobin, white blood cells, IgG (total and IgG4), international normalized ratio – (dates, values, normal ranges and value flags for each test)

BMI, body mass index; ERCP, endoscopic retrograde cholangio-pancreatography; FISH, fluorescent in situ hybridization; MRE, magnetic resonance elastography; IBD, inflammatory bowel disease; UC, ulcerative colitis; CD, Crohn’s disease; UDCA, ursodeoxycholic acid; EMR, electronic medical record

### Questionnaires

Following study enrollment each participant is asked to fill out a custom-designed, comprehensive questionnaire, the Liver Biobank Questionnaire, which collects scientifically relevant demographic, clinical, medical, surgical, dietary, environmental exposure, occupational, and employment information from PSC patients. We have previously published a large study of environmental factors in PSC using the original version of this questionnaire [22]. We have recently (October 14th, 2020) updated and modified this questionnaire to better evaluate past and current medications, which now includes versions that can be completed online or using a smartphone. A paper version of the current Liver Biobank Questionnaire is provided as Additional file 1. Participants in the current study will be asked to complete the new questionnaire at 2-year intervals to help in documenting changes in PSC patients’ lifestyles, exposures to medications and identify new PSC-related outcomes that are not part of the Mayo Clinic medical record. Patients enrolling in the current protocol are also asked to fill out a 24-hour Food Frequency Questionnaire in conjunction with submission of their stool sample, which is available online via the Automated Self-Administered 24-hour Dietary Assessment Tool (https://www.asa24.nci.nih.gov/researchersite/) .

### Biospecimens

Following enrollment, patients are asked to provide biospecimens, which are collected using mail-in kits prepared and distributed by the Mayo Clinic Biospecimens Accessioning and Processing (BAP) lab located in Rochester, Minnesota. These kits can be processed by the patients’ home clinic phlebotomy lab (blood samples) or in the comfort of their home (urine and stool samples) and are returned via overnight carrier service to the BAP lab. Once received, this lab coordinates sample transfers and provides all required sample preparation services. The previous protocol primarily collected blood, which was processed and stored as aliquots of DNA, buffy coat, PBMC, plasma and serum. Additionally, this blood sample was used to run a small panel of liver biochemistries using the General Clinical Laboratory of Mayo Clinic: alkaline phosphatase, alanine aminotransferase, aspartate aminotransferase, total bilirubin, and albumin as a prospective measure of disease activity at time of sample collection. Later, stool samples were also requested and collected from a subset of patients enrolled in the previous protocol. Participants in the previous protocol were offered renumeration in the form of a choice of health-related books.

In the current protocol, we ask participants to provide blood, urine, and stool samples at time of enrollment. While we encourage individuals to provide all sample types, this is not required. The current blood kit collects 48 ml of blood, which is processed and stored as aliquots of DNA, buffy coat, STRECK-tube platelet-poor plasma (for cell-free DNA), PAXGENE tube blood (for RNA), PBMC, plasma and serum. Additionally, an expanded panel of liver biochemistries adding hematocrit, hemoglobin, mean corpuscular volume, platelets, sodium and complete blood count is run at time of sample collection, to assist evaluation of disease state and provide additional data for use in analysis of -omics datasets. For PSC patients, we plan to request additional blood, urine, and stool samples at 2-to-4-year intervals, depending on level of interest. However, agreement to provide future samples is not a requirement for enrollment. Patients enrolled in the current protocol are offered renumeration in the form of $40.00 US per submitted sample.

In addition to samples collected via mail-in kits, we collect bile and biliary brushings from participants during clinically scheduled endoscopic retrograde cholangio-pancreatography (ERCP) at Mayo Clinic in Rochester, MN. Bile aspirated during ERCP is collected in sterile tubes and placed on ice after collection for processing and storage. Also, biliary brushings obtained during ERCP are collected in sterile containers containing Phosphate-buffered saline solution, placed on ice, and used immediately or stored. Finally, fresh tissue specimens (liver parenchyma and bile duct) are collected through the Mayo Clinic Tissue Request Acquisition Group from patients with PSC and controls who undergo liver transplantation, partial hepatectomy, or are a liver donor at Mayo Clinic.

All biospecimens are stored de-identified, labeled with a study-ID traceable only by the team of the principle investigator (PI) or by certain members of the BAP lab staff. The majority of biospecimens collected under the previous IRB protocol, as well as all bile and tissue samples, are stored in the laboratory of the PI in access-restricted freezers. In contrast, most of the biospecimens collected under the current protocol are stored in the BAP lab following sample preparation. This storage service provides improved back-up and monitoring service and facilitates transfer to core laboratories responsible for aliquoting samples and performing omics-scale experiments. Sample locations and information for BAP lab-resident samples are tracked using BAP’s custom research laboratory information management system available to BAP lab staff. Additionally, all study samples, whether stored in the PI lab or the BAP lab, are tracked by study staff using a customized database that coordinates sample location, sample usage, prospective lab results and mapping to phenotypic and clinical data.

### Data and specimen sharing

The data and biospecimens generated and obtained by the PSC-SCR will be shared with interested researchers proposing scientifically sound PSC-focused research and with the broader research community to the extent possible. As sample availability is limited, projects proposing -omics scale interrogations focussed on PSC will be prioritized, although such scale is not required. Due to the complex and evolving nature of the resource, datasets and samples will be prepared and provided on a case-by-case basis in consult with requesting investigators. This approach will help to ensure specific research questions can be adequately addressed, ethics considerations undergo proper review and that data and sample usage is agreed upon and documented. The expectation is that data generated using PSC-SCR samples will be made fully available to the PSC-SCR in timely fashion, respecting the ability of requesting investigators to independently publish.

### Status of the PSC scientific community resource

As of March 1, 2021, a total of 1396 PSC patients and 1,352 controls have been enrolled in the PSC-SCR and provided blood specimens. This includes 1073 patients only enrolled in our previous IRB protocol, 222 patients only enrolled in our current protocol and 101 patients enrolled in both protocols. Among the controls, 642 are only enrolled in our previous protocol, 702 only in the current protocol and 8 in both. An additional 188 PSC patients and 529 controls have enrolled in our current protocol but have not yet provided their blood specimen, and thus, are not described in this manuscript.

Relevant clinical and demographic data as well as current sample availability for all participants in the previous IRB protocol is presented in Table 2. Demographics of the patient group are consistent with previous reports as 62.4% are male, median age of diagnosis is 40.6 years and 77.2% have concurrent IBD. Median age at study entry was 51.6 years with median disease duration of 6.2 years. In contrast, the controls are older with a median age at study entry of 61.3 years and only 27.2% were male. This difference is due to this control population being shared with a similar biobank of PBC patients that we maintain, of which 90% of the patients are female and they tend to be older than PSC patients. As well, the number of controls collected under the previous protocol is smaller than the patient population due to limited resources available to support collection of controls at that time. Clinical follow-up after sample collection is available for 52.0% of the patient population, with 19% having over 6 years of follow-up. The 48.0% of patients without follow-up include all patients recruited through our collaborators (n = 437) as well as self-referred (n = 17) and Mayo Clinic patients (n = 110) with no available medical records after study entry. A total of 366 patients had advanced disease at time of study enrollment, 243 of whom had received a liver transplant. An additional 107 patients developed advanced disease and 74 received a liver transplant during follow-up. Moreover, 76 of the patients were diagnosed with hepatobiliary cancer prior to study entry, with 50 patients developing hepatobiliary cancer during follow-up. Genomic DNA and/or buffy coat, plasma and PBMC samples are available for the majority (> 90%) of PSC patients and controls while serum is more limited as it was collected as a residual of prospective liver function tests in the previous protocol. Finally, stool samples were not collected from controls, but are available for 95 of the patients from the previous protocol.

**Table 2:** PSC patients and controls enrolled in the previous protocol (IRB #670-02)

Variable	PSC patients	Controls
*n*	1174	650
*Age (yrs)*^*a*^, median (IQR)	51.6 (36.6–61.6)	61.3 (53.9–68.1)
*Sex*, n (%) male	733 (62.4)	177 (27.2)
*Race*, n (%) Caucasian	1087 (92.6)	646 (99.4)
*Available Specimens*, n (%) of participants [total # of aliquots]		
Genomic DNA / BC	1166 (99.3) [3,316]	624 (96.0) [1,723]
Plasma	1117 (95.1) [5,821]	635 (97.7) [2,100]
Serum	719 (61.2) [1,705]	540 (83.1) [1,229]
PBMC	1091 (92.9) [2,743]	607 (93.4) [1,120]
Stool	95 (8.1) [185]	not collected
*Age at PSC dx (yrs)*, median (IQR)	40.6 (29.0-52.1)	na
*PSC duration (yrs)*^*a*^, median (IQR)	6.2 (2.3–12.9)	na
*IBD Type*, n (%) of patients		
Ulcerative colitis	728 (62.4)	na
Crohn’s Disease	119 (10.2)	
Indeterminate IBD	54 (4.6)	
No IBD	266 (22.8)	
Status unknown	7	
*Clinical follow-up*^*b*^, n (%) of patients		
None	564 (48.0)	na
0–3 Years	180 (15.3)	
3–6 Years	207 (17.6)	
6 + Years	223 (19.0)	
*Liver Transplant*, n (%) of patients		
Prior to enrollment	243 (20.7)	na
In follow-up	74 (6.3)	
No transplant	857 (73.0)	
*Advanced Disease*^*c*^, n (%) of patients		
Prior to enrollment	366 (31.2)	na
In follow-up	107 (9.1)	
No advanced disease	701 (59.7)	
*Hepatobiliary Cancer*^*d*^, n (%) of patients		
Prior to enrollment	76 (6.5)	na
In follow-up	50 (4.3)	
No hepatobiliary cancer	1048 (89.3)	

na, not applicable; BC, buffy coat; PBMC, peripheral blood mononuclear cells; IBD, inflammatory bowel disease

^a^At time of blood sample collection

^b^Clinical follow-up post first blood sample collection performed under IRB#670-02

^c^Advanced disease defined as having one or more of the following: liver transplant, cirrhosis determination or hepatic decompensation event

^d^Hepatobiliary cancer defined as having one or more of the following: cholangiocarcinoma, hepatocellular carcinoma or gallbladder cancer

The clinical and demographic data for all participants in the current IRB protocol that have provided their blood specimen is presented in Table 3. As with the previous protocol, the demographics of the patient group are consistent with other reports with 56.0% being male, median age of diagnosis of 41.5 years and 78.0% having concurrent IBD. The current median age at study entry is 52.6 years with median disease duration of 6.1 years, which is similar to the previous study. Again, the control population is shared with our ongoing biobank of PBC patients and thus are older with a median age at study entry of 67.5 years and only 31.8% are male. However, unlike the previous protocol, we are now able to dedicate additional resources to collecting controls in order to better perform the planned multi-omic studies and we currently have over twice as many controls as patients. Clinical follow-up after sample collection is available for 68.1% of the patient population, the majority having 0–3 years of follow-up as the current study has only been enrolling for ~ 4 years. Unlike the prior protocol, many of the patients with no follow-up have only recently enrolled in the study and we anticipate having better access to medical records such that 80–90% of the cohort is likely to have meaningful follow-up in the future. Due to the nature of the planned research, recruitment efforts in the current protocol primarily focus on pre-transplant patients. Thus, while 87 of the patients had advanced disease at time of study enrollment only 9 of them had already received a liver transplant. So far, an additional 20 patients developed advanced disease and 24 patients received a liver transplant during follow-up. A total of 22 patients were diagnosed with hepatobiliary cancer prior to study entry, with an additional 7 patients developing hepatobiliary cancer during follow-up. Samples from the blood collection kit (i.e., genomic DNA and/or buffy coat, plasma, serum, PBMC, RNA and plasma cell-free DNA) are available for most of the PSC patients and controls, those without samples being due to rare problems with collection such as delivery delays putting samples outside of quality control standards. Stool and urine samples have been provided by over 95% of controls; however, fewer patients provide those sample type (75.8% stool samples and 83.3% urine samples) likely because many patients have concurrent IBD. Finally, we have, thus far, collected bile samples from 34 patients during ERCP and liver tissue from 18 patients at time of liver transplant.

**Table 3:** PSC patients and controls enrolled in the current protocol (IRB #16-005892)

Variable	PSC patients	Controls
*n*	323	710
*Age (yrs)*^*a*^, median (IQR)	52.6 (36.7–65.1)	67.5 (58.3–73.3)
*Sex*, n (%) male	181 (56.0)	226 (31.8)
*Race*^*b*^, n (%) Caucasian	311 (96.3)	693 (98.3)
*Available specimens*, n (%) of participants [total # of aliquots]		
Genomic DNA / BC	318 (98.5) [646]	706 (99.4) [1,409]
Plasma	318 (98.5) [1,273]	706 (99.4) [2,736]
Serum	316 (97.8) [1,143]	705 (99.3) [2,432]
PBMC	317 (98.1) [967]	706 (99.4) [2,118]
RNA (whole blood)	318 (98.5) [647]	705 (99.3) [1,410]
Cell-free DNA (plasma)	315 (97.5) [321]	703 (99.0) [703]
Stool	245 (75.8) [733]	678 (95.5) [2,034]
Urine	269 (83.3) [1,224]	676 (95.2) [3,131]
Bile	34 (10.5) [128]	na
Liver tissue	18 (5.6) [107]	na
*Age at PSC dx (yrs)*, median (IQR)	41.5 (28.8–54.3)	na
*PSC duration (yrs)*^*a*^, median (IQR)	6.1 (2.1–12.6)	na
*IBD type*, n (%) of patients		
Ulcerative colitis	203 (62.8)	na
Crohn’s Disease	43 (13.3)	
Indeterminate IBD	6 (1.9)	
No IBD	71 (22.0)	
*Clinical follow-up*^*c*^, n (%) of patients		
None	103 (31.9)	na
0–3 Years	217 (67.2)	
3–6 Years	3 (0.9)	
6 + Years	0	
*Liver transplant*, n (%) of patients		
Prior to enrollment	9 (2.8)	na
In follow-up	24 (7.4)	
No liver transplant	290 (89.8)	
*Advanced disease*^*d*^, n (%) of patients		
Prior to enrollment	87 (26.9)	na
In follow-up	20 (6.2)	
No advanced disease	216 (66.9)	
*Hepatobiliary Cancer*^*e*^, n (%) of patients		
Prior to enrollment	22 (6.8)	na
In follow-up	7 (2.2)	
No hepatobiliary cancer	294 (91.0)	

na, not applicable; BC, buffy coat; PBMC, peripheral blood mononuclear cells; IBD, inflammatory bowel disease

^a^At time of blood sample collection

^b^Race information not available for five controls

^c^Clinical follow-up post first blood sample collection performed under IRB#16-005892

^d^Advanced disease defined as having one or more of the following: liver transplant, cirrhosis determination or hepatic decompensation event

^e^Hepatobiliary cancer defined as having one or more of the following: cholangiocarcinoma, hepatocellular carcinoma or gallbladder cancer

## Discussion

Advancements in understanding the complex pathogenesis of PSC are needed to improve treatment options and guidance in order to achieve better outcomes for PSC patients. Patient-based studies leveraging the latest technologies for targeted and wide-scale interrogation of multiple omics layers offer promise to accelerate PSC research through discovery of unappreciated aspects of disease pathogenesis. However, the rarity of PSC severely limits such studies. Here we describe our effort to overcome this limitation, the PSC-SCR, a repository of patient biospecimens coupled with clinical and omics data for use by the broader PSC research community.

The PSC-SCR is a continuation of our past efforts in the study of PSC spanning over 15 years, which contributed significantly to our understanding of genetic [19] and environmental [22] components of disease and led to development of a novel score for predicting the likelihood of future hepatic decompensation in PSC patients [25]. The resource is currently being utilized to perform omics-scale projects investigating aspects of the exposome, metabolome, methylome, immunome and microbiome in PSC. We anticipate these projects will provide novel insight into disease pathogenesis that will lead to new lines of research across the spectrum of potentially important disease mechanisms.

While it is a promising resource for PSC research, there are limitations to the PSC-SCR. First, the primary goal of the previous protocol was to perform genome wide association studies, which only require a confirmed PSC diagnosis. Thus, many of the patients from this protocol lack extensive phenotyping and clinical follow-up needed for studies aimed to identify new predictive biomarkers. Second, Mayo Clinic is a major referral center for patients who develop PSC-related complications (advanced disease, abnormal biliary cytology, suspicious strictures, and biopsy-proven CCA). As a result, a sizeable proportion of the patients invited to participate in our protocols may represent the more severe end of the disease spectrum. Third, previous efforts have only collected samples at a single time point. Thus, the number of patients developing disease endpoints within a reasonable time frame for event prediction is limited. To overcome this limitation, we plan to begin collecting serial specimens at regular intervals from interested patients enrolled in the current protocol. Finally, incomplete medical records, lack of adequate follow-up, and lack of precise information on disease diagnosis, endpoints and outcomes is a major limitation, particularly for enrolled patients who are seen and cared for at outside institutions. To mitigate this limitation, we are starting to implement use of emerging tools for accessing electronic medical records from external sites in order to more completely capture this important information.

In summary, the PSC-SCR seeks to utilize state-of-the-art technology to generate -omics scale data from a large cohort of patients with PSC. The generated data and residual biospecimens will be made available to the broader PSC research community to the extent possible and will serve as a new platform to further our understanding of PSC etiopathogenesis that could provide the framework for future drug discovery in PSC.

## Supplementary Information

**Additional file 1**. Liver Biobank Questionnaire, which collects scientifically relevant information. Subjects are asked to fill out the questionnaire at the time of enrollment and every 2 years.

## Data Availability

The datasets and biospecimens described in this manuscript will be shared with interested researchers proposing scientifically sound PSC-focused research on reasonable request and with approval of the PI and appropriate review boards. Request for data can be submitted by email to Konstantinos N. Lazaridis, MD at (lazaridis.konstantinos@mayo.edu).

## Abbreviations

BAP: Biospecimens accessioning and processing facility
CCA: Cholangiocarcinoma
ERCP: Endoscopic retrograde cholangio-pancreatography
PBMC: Peripheral blood mononuclear cells
PI: Principle investigator
PSC: Primary sclerosing cholangitis
PSC-SCR: PSC Scientific Community Resource
PSC-SDMS: PSC study data management system
