# Supplementary material for: The PSC scientific community resource: an asset for multi-omics interrogation of primary sclerosing cholangitis
Source: BMC Gastroenterol. 2021 Sep 25;21:353. doi: 10.1186/s12876-021-01930-2 (PMC8465725; doi:10.1186/s12876-021-01930-2)
Supplement: Supplementary file 1 — Additional file 1. Liver Biobank Questionnaire, which collects scientifically relevant information. Subjects are asked to fill out the questionnaire at the time of enrollment and every 2 years. [file 12876_2021_1930_MOESM1_ESM.pdf]

MAYO
CLINIC

Liver Biobank Questionnaire

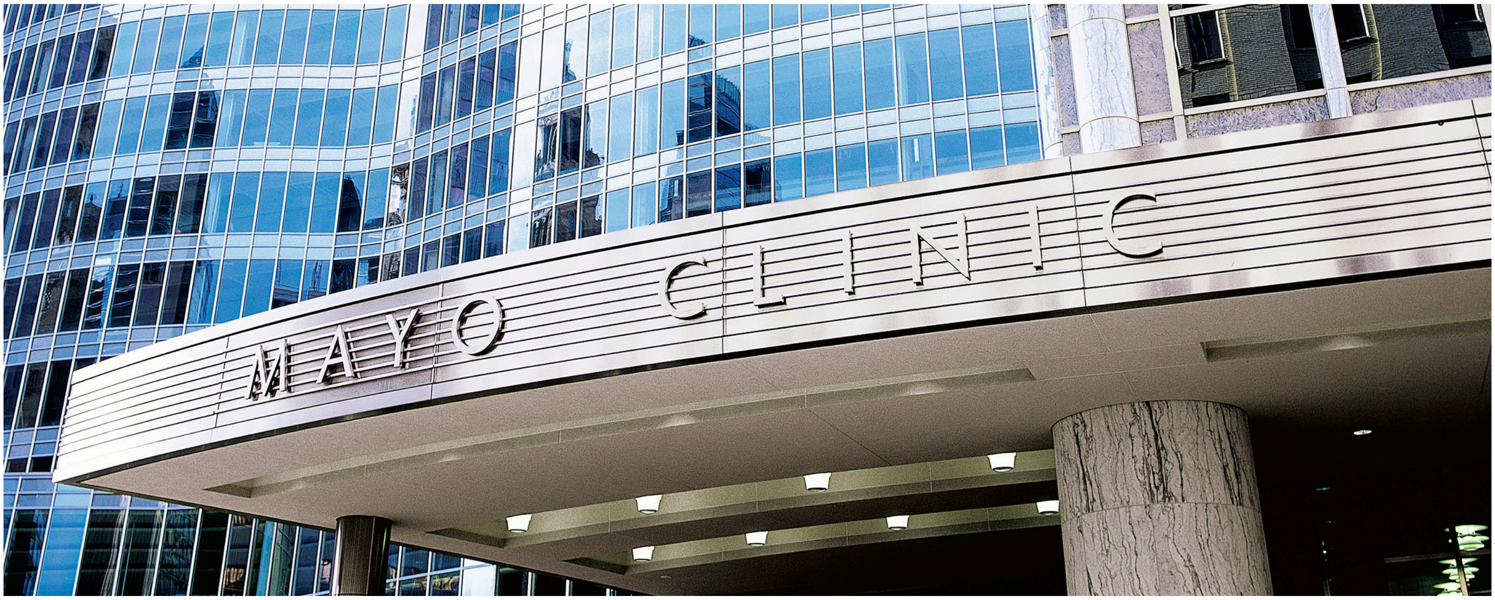

Survey Research Center

Clinic Number
Name
Address
Phone Number

Please enter above any missing information or change any that is incorrect.

INSTRUCTIONS: PLEASE CHECK THE APPROPRIATE BOX OR FILL IN THE BLANK AS INDICATED.

1. **Today's Date:** __ __/__ __/__ __ __ __
   Month Day Year

2. **What is your current height?** (Please record your response in feet and inches.)

   __ Feet __ __ Inches

3. **What is your current weight?** (Please round to the nearest whole number.)

   __ __ __ Pounds

4. **How do you describe yourself?** (Mark all that apply.)

   1 ☐ Female
   1 ☐ Male
   1 ☐ Transgender
   1 ☐ Do not identify as female, male, or transgender

5. **Are you currently:**

   1 ☐ Married
   2 ☐ Separated
   3 ☐ Divorced
   4 ☐ Widowed
   5 ☐ Never been married
   6 ☐ In a relationship where you are currently living together as if you are married

6. **Which of the following best describes you?**

1 ☐ Working full-time for pay (more than 35 hours a week)
2 ☐ Working part-time for pay
3 ☐ Not working for pay

If not working for pay, are you: (Mark one.)

1 ☐ Full-time homemaker
2 ☐ Seasonal worker
3 ☐ Disabled
4 ☐ Retired
5 ☐ In school
6 ☐ Unemployed/looking for work
7 ☐ Other, please specify: ______________________________

7. **What is the nature of the business or industry where you have worked during the majority of your life?** (Mark one.)

1 ☐ Active duty military
2 ☐ Mining
3 ☐ Construction
4 ☐ Public administration
5 ☐ Farming, forestry, fishing, and hunting
6 ☐ Retail trade
7 ☐ Finance, insurance, real estate, and rental and leasing
8 ☐ Information and communication
9 ☐ Services: Arts, entertainment, recreation, accommodations, and food
10 ☐ Services: Education, health, and social
11 ☐ Services: Professional, scientific, management, and administrative
12 ☐ Services: Waste management
13 ☐ Services: Other, specify: ______________________________
14 ☐ Telecommunications
15 ☐ Transportation and warehousing
16 ☐ Utilities
17 ☐ Wholesale trade
18 ☐ Manufacturing/production
19 ☐ Other, specify: ______________________________
20 ☐ None of the above

8. **From birth to 18 years of age, have you lived:** (Mark all that apply.)

1 ☐ On a working farm or ranch
2 ☐ In a rural home or hobby farm, not a working farm or ranch
3 ☐ In a suburb, city, or village

9. **Where do you currently live most of the year?** (Mark one.)

1 ☐ On a working farm or ranch
2 ☐ In a rural home or hobby farm, not a working farm or ranch
3 ☐ In a suburb, city, or village

10. **Have you ever worked on a working farm?**

1 ☐ Yes 2 ☐ No

What type of farm was it?

1 ☐ Commercial
2 ☐ Dairy
3 ☐ Cattle
4 ☐ Agricultural

11. **For the job (includes homemaking) you have held the longest, approximately how much of the time were you engaged in each of the following physical activities?**

	None of the time	A little of the time	Some of the time	Most of the time	All of the time
**Sitting**	1 ☐	2 ☐	3 ☐	4 ☐	5 ☐
**Walking**	1 ☐	2 ☐	3 ☐	4 ☐	5 ☐
**Light manual labor**	1 ☐	2 ☐	3 ☐	4 ☐	5 ☐
**Heavy manual labor**	1 ☐	2 ☐	3 ☐	4 ☐	5 ☐

12. **On average, how many times a day do you eat high-fat food such as red meat, fried food, whole milk, regular cheese, ice cream, baked goods, or regular dressing?** (Mark one.)

1 ☐ 0 to 1
2 ☐ 2
3 ☐ 3 or more

13. **How many servings of fruit do you eat in a typical day?** (One serving: 1 medium piece of fruit or ¾ cup of fruit juice.)

1 ☐ 0 to 1 serving
2 ☐ 2 servings
3 ☐ 3 servings
4 ☐ 4 servings
5 ☐ 5 servings

14. **How many servings of vegetables do you eat in a typical day?** (One serving: 1 cup raw leafy vegetables, ½ cup cooked vegetables, or ¾ cup vegetable juice.)

1 ☐ 0 to 1 serving
2 ☐ 2 servings
3 ☐ 3 servings
4 ☐ 4 servings
5 ☐ 5 servings

15. **Approximately how much calcium do you have in a typical day (include food and supplements)?**

1 ☐ Less than 600 mg
2 ☐ 600 to 1,200 mg
3 ☐ More than 1,200 mg

16. **How many servings of diet soft drinks do you have in a typical day?** (Average size is 1 can or 12 ounce glass.)

1 ☐ None **— Skip to question 17**

2 ☐ 1 to 2 servings
3 ☐ 3 to 4 servings
4 ☐ 5 to 6 servings
5 ☐ 7 to 9 servings
6 ☐ 10 or more servings

→ **How often is the diet soft drink you drink decaffeinated?**

1 ☐ Never or almost never
2 ☐ About ¼ of the time
3 ☐ About ½ of the time
4 ☐ About ¾ of the time
5 ☐ Always or almost always

17. How many servings of regular (non-diet) soft drinks do you have in a typical day? (Average size is 1 can or 12 ounce glass.)

1 ☐ None **— Skip to question 18**

2 ☐ 1 to 2 servings
3 ☐ 3 to 4 servings
4 ☐ 5 to 6 servings
5 ☐ 7 to 9 servings
6 ☐ 10 or more servings

→ **How often is the regular (non-diet) soft drink you drink decaffeinated?**

1 ☐ Never or almost never
2 ☐ About ¼ of the time
3 ☐ About ½ of the time
4 ☐ About ¾ of the time
5 ☐ Always or almost always

18. How many cups of coffee, caffeinated or decaffeinated, do you drink in a typical day?

1 ☐ None **— Skip to question 19**

2 ☐ Less than 1 cup each month
3 ☐ 1 cup per week
4 ☐ 2 to 4 cups per week
5 ☐ 5 to 6 cups per week
6 ☐ 1 cup per day
7 ☐ 2 to 3 cups per day
8 ☐ 4 to 5 cups per day
9 ☐ 6 or more cups per day

→ **How often is the coffee you drink decaffeinated?**

1 ☐ Never or almost never
2 ☐ About ¼ of the time
3 ☐ About ½ of the time
4 ☐ About ¾ of the time
5 ☐ Always or almost always

19. How many cups of tea, caffeinated or decaffeinated, do you drink in a typical day?

1 ☐ None **— Skip to question 20**

2 ☐ Less than 1 cup each month
3 ☐ 1 cup per week
4 ☐ 2 to 4 cups per week
5 ☐ 5 to 6 cups per week
6 ☐ 1 cup per day
7 ☐ 2 to 3 cups per day
8 ☐ 4 to 5 cups per day
9 ☐ 6 or more cups per day

→ **How often is the tea you drink decaffeinated?**

1 ☐ Never or almost never
2 ☐ About ¼ of the time
3 ☐ About ½ of the time
4 ☐ About ¾ of the time
5 ☐ Always or almost always

20. Have you used any of these tobacco products for 12 months or longer?
(Mark a response for each tobacco product.)

	Yes	No	If you have used this tobacco product for 12 months or longer, how many years did you use it?
**Cigar**	1 ☐	2 ☐	__ __ Years
**Pipe**	1 ☐	2 ☐	__ __ Years
**Snuff**	1 ☐	2 ☐	__ __ Years
**Chewing tobacco**	1 ☐	2 ☐	__ __ Years

21. Have you smoked at least 100 cigarettes in your entire life?

1 ☐ Yes   2 ☐ No

How old were you when you first started smoking cigarettes on a regular basis?

__ __ Age

On average, how many cigarettes do/did you smoke per day?

1 ☐ 1 to 10 per day
2 ☐ 11 to 20 per day
3 ☐ 21 to 30 per day
4 ☐ 31 to 40 per day
5 ☐ 41 or more per day

Do you currently smoke cigarettes?

1 ☐ Yes   2 ☐ No

What year did you quit (e.g., 2020)?

__ __ __ __ Year

22. Did you ever live in a household with someone who smoked cigarettes regularly while in your presence?

1 ☐ Yes 2 ☐ No

For how many years altogether did you live in a household with someone who smoked cigarettes regularly in your presence?

__ __ Years

Indicate the amount of secondhand exposure per day by the approximate number of cigarettes or packs smoked by the person(s) from your household.

1 ☐ 1 to 10 cigarettes (up to ½ pack)
2 ☐ 11 to 20 cigarettes (½ to 1 pack)
3 ☐ 21 to 40 cigarettes (1 to 2 packs)
4 ☐ 41 to 60 cigarettes (2 to 3 packs)
5 ☐ More than 60 cigarettes (3 packs or more)

What age(s) were you exposed to secondhand smoke from your household? (Mark all that apply.)

1 ☐ Younger than 5
1 ☐ 5 to 9
1 ☐ 10 to 19
1 ☐ 20 to 29
1 ☐ 30 to 39
1 ☐ 40 to 49
1 ☐ 50 to 59
1 ☐ 60 to 69
1 ☐ 70 to 79
1 ☐ 80 and older

23. Did you ever work in an area where others smoked regularly in your presence?

1 ☐ Yes 2 ☐ No

For how many years altogether was this the case?

__ __ Years

Indicate the amount of secondhand exposure per day by the approximate number of cigarettes or packs smoked by the person(s) from your workplace.

1 ☐ 1 to 10 cigarettes (up to ½ pack)
2 ☐ 11 to 20 cigarettes (½ to 1 pack)
3 ☐ 21 to 40 cigarettes (1 to 2 packs)
4 ☐ 41 to 60 cigarettes (2 to 3 packs)
5 ☐ More than 60 cigarettes (3 packs or more)

What age(s) were you exposed to secondhand smoke from your workplace? (Mark all that apply.)

1 ☐ Younger than 16
1 ☐ 16 to 19
1 ☐ 20 to 29
1 ☐ 30 to 39
1 ☐ 40 to 49
1 ☐ 50 to 59
1 ☐ 60 to 69
1 ☐ 70 to 79
1 ☐ 80 or older

24. Are you currently regularly taking (2 times or more per week for at least 3 months), or have you recently (not currently, but during the past 12 months) taken any of the following over-the-counter vitamins/supplements? (Mark all that apply.)

	Currently **taking (2 times or more per week for at least 3 months)**	*Recently* **(not currently, but during the past 12 months)**
**Multivitamins**	1 ☐	1 ☐
**Prenatal vitamins**	1 ☐	1 ☐
**Vitamin A**	1 ☐	1 ☐
**Vitamin B**	1 ☐	1 ☐
**Vitamin C**	1 ☐	1 ☐
**Vitamin D**	1 ☐	1 ☐
**Vitamin E**	1 ☐	1 ☐
**Beta carotene**	1 ☐	1 ☐
**Calcium**	1 ☐	1 ☐
**Folate**	1 ☐	1 ☐
**Iron**	1 ☐	1 ☐
**Selenium**	1 ☐	1 ☐
**Zinc**	1 ☐	1 ☐
**5-HTP**	1 ☐	1 ☐
**Acidophilus**	1 ☐	1 ☐
**Bee pollen or royal jelly**	1 ☐	1 ☐
**Chondroitin**	1 ☐	1 ☐
**CoQ10**	1 ☐	1 ☐
**DHEA**	1 ☐	1 ☐
**Fiber supplement (Metamucil®, etc.)**	1 ☐	1 ☐
**Fish oil/omega fatty acids/EPA/DHA)**	1 ☐	1 ☐
**Glucosamine**	1 ☐	1 ☐
**Melatonin**	1 ☐	1 ☐
**Progesterone cream**	1 ☐	1 ☐
**SAM-e**	1 ☐	1 ☐
**Xenadrine™**	1 ☐	1 ☐
**Other, specify:** ____________	1 ☐	1 ☐
**Other, specify:** ____________	1 ☐	1 ☐
**Other, specify:** ____________	1 ☐	1 ☐
**None**	1 ☐	1 ☐

25. In the past 4 weeks, have you used any:

Probiotics:

1 ☐ Yes 2 ☐ No 3 ☐ Don't know

If you used probiotics in the past 4 weeks, what is the name of the probiotic(s)?

____

Antibiotics:

1 ☐ Yes 2 ☐ No 3 ☐ Don't know

If you used antibiotics in the past 4 weeks, what is the name of the antibiotic(s)?

____

Steroid pills or injections:

1 ☐ Yes 2 ☐ No 3 ☐ Don't know

If you used steroid pills or injections in the past 4 weeks, what is the name of the steroid pill or injection?

____

26. Considering a 7-day period (1 week), how many times on average do you exercise more than 15 minutes during your free time?

	None	1 time	2 times	3 times	4 times	5 times	6 times	7 times	8 or more times
**Strenuous exercise** **(heart beats rapidly)** (i.e., running, jogging vigorous swimming, etc.) .	1 ☐	2 ☐	3 ☐	4 ☐	5 ☐	6 ☐	7 ☐	8 ☐	9 ☐
**Moderate exercise** **(not exhausting)** (i.e., fast walking, swimming, etc.)	1 ☐	2 ☐	3 ☐	4 ☐	5 ☐	6 ☐	7 ☐	8 ☐	9 ☐
**Mild exercise** **(minimal effort)** (i.e., easy walking, archery, bowling, etc.) . . .	1 ☐	2 ☐	3 ☐	4 ☐	5 ☐	6 ☐	7 ☐	8 ☐	9 ☐

27. **How often did you have a drink containing alcohol in the past 12 months?** (Consider a "drink" to be a can or bottle of beer, a glass of wine, a wine cooler, 1 cocktail, or a shot of liquor, i.e., scotch, gin, or vodka.)

1 ☐ Never **— Skip to question 28**

2 ☐ Once a month or less
3 ☐ 2 to 4 times a month
4 ☐ 2 to 3 times a week
5 ☐ 4 to 5 times a week
6 ☐ 6 or more times a week

→ **How many drinks did you have on a typical day when you were drinking in the past 12 months?**

1 ☐ 0 to 2 drinks
2 ☐ 3 to 4 drinks
3 ☐ 5 to 6 drinks
4 ☐ 7 to 9 drinks
5 ☐ 10 or more drinks

How often did you have 6 or more drinks on one occasion in the past 12 months?

1 ☐ Never
2 ☐ Less than monthly
3 ☐ Monthly
4 ☐ Weekly
5 ☐ Daily or almost daily

28. **Which of the following medications have you taken?** (If you have taken a medication, please provide the dose, the frequency, indication [why you are taking], and the most recent date you started or stopped the medication [if you do not know the exact date, please enter what you do know or leave it blank].)

Drug used for Primary Biliary Cholangis (PBC)/Primary Sclerosing Cholangitis (PSC)

1 ☐ Ursodiol
Dose: ________________ Frequency: ________ 1 ☐ Per day 2 ☐ Per week 3 ☐ Per month
Indication (reason): ____
Date started: __ __/__ __/__ __ __ __ (Month Day Year) OR Date stopped: __ __/__ __/__ __ __ __ (Month Day Year)

1 ☐ Obeticholic acid (OCALIVA®)
Dose: ________________ Frequency: ________ 1 ☐ Per day 2 ☐ Per week 3 ☐ Per month
Indication (reason): ____
Date started: __ __/__ __/__ __ __ __ (Month Day Year) OR Date stopped: __ __/__ __/__ __ __ __ (Month Day Year)

Beta Blockers

1 ☐ Propranolol
Dose: ________________ Frequency: ________ 1 ☐ Per day 2 ☐ Per week 3 ☐ Per month
Indication (reason): ____
Date started: __ __/__ __/__ __ __ __ (Month Day Year) OR Date stopped: __ __/__ __/__ __ __ __ (Month Day Year)

Beta Blockers (continued)

1 ☐ Nadolol

Dose: ________________ Frequency: ________ 1 ☐ Per day 2 ☐ Per week 3 ☐ Per month

Indication (reason): ____

Date started: __ __/__ __/__ __ __ __ (Month Day Year) OR Date stopped: __ __/__ __/__ __ __ __ (Month Day Year)

1 ☐ Carvedilol

Dose: ________________ Frequency: ________ 1 ☐ Per day 2 ☐ Per week 3 ☐ Per month

Indication (reason): ____

Date started: __ __/__ __/__ __ __ __ (Month Day Year) OR Date stopped: __ __/__ __/__ __ __ __ (Month Day Year)

Hepatic Encephalopathy

1 ☐ Rifaximin (Xifaxan®)

Dose: ________________ Frequency: ________ 1 ☐ Per day 2 ☐ Per week 3 ☐ Per month

Indication (reason): ____

Date started: __ __/__ __/__ __ __ __ (Month Day Year) OR Date stopped: __ __/__ __/__ __ __ __ (Month Day Year)

1 ☐ Lactulose

Dose: ________________ Frequency: ________ 1 ☐ Per day 2 ☐ Per week 3 ☐ Per month

Indication (reason): ____

Date started: __ __/__ __/__ __ __ __ (Month Day Year) OR Date stopped: __ __/__ __/__ __ __ __ (Month Day Year)

1 ☐ Neomycin

Dose: ________________ Frequency: ________ 1 ☐ Per day 2 ☐ Per week 3 ☐ Per month

Indication (reason): ____

Date started: __ __/__ __/__ __ __ __ (Month Day Year) OR Date stopped: __ __/__ __/__ __ __ __ (Month Day Year)

Diuretics

1 ☐ Lasix

Dose: ________________ Frequency: ________ 1 ☐ Per day 2 ☐ Per week 3 ☐ Per month

Indication (reason): ____

Date started: __ __/__ __/__ __ __ __ (Month Day Year) OR Date stopped: __ __/__ __/__ __ __ __ (Month Day Year)

1 ☐ Bumetanide

Dose: ________________ Frequency: ________ 1 ☐ Per day 2 ☐ Per week 3 ☐ Per month

Indication (reason): ____

Date started: __ __/__ __/__ __ __ __ (Month Day Year) OR Date stopped: __ __/__ __/__ __ __ __ (Month Day Year)

Diuretics (continued)

1 ☐ Spironolactone

Dose: ________________ Frequency: ________ 1 ☐ Per day 2 ☐ Per week 3 ☐ Per month

Indication (reason): ____

Date started: __ __/__ __/__ __ __ __ OR Date stopped: __ __/__ __/__ __ __ __
Month Day Year Month Day Year

1 ☐ Amiloride

Dose: ________________ Frequency: ________ 1 ☐ Per day 2 ☐ Per week 3 ☐ Per month

Indication (reason): ____

Date started: __ __/__ __/__ __ __ __ OR Date stopped: __ __/__ __/__ __ __ __
Month Day Year Month Day Year

1 ☐ Triamterene

Dose: ________________ Frequency: ________ 1 ☐ Per day 2 ☐ Per week 3 ☐ Per month

Indication (reason): ____

Date started: __ __/__ __/__ __ __ __ OR Date stopped: __ __/__ __/__ __ __ __
Month Day Year Month Day Year

1 ☐ Metolazone

Dose: ________________ Frequency: ________ 1 ☐ Per day 2 ☐ Per week 3 ☐ Per month

Indication (reason): ____

Date started: __ __/__ __/__ __ __ __ OR Date stopped: __ __/__ __/__ __ __ __
Month Day Year Month Day Year

1 ☐ Hydrochlorothiazide

Dose: ________________ Frequency: ________ 1 ☐ Per day 2 ☐ Per week 3 ☐ Per month

Indication (reason): ____

Date started: __ __/__ __/__ __ __ __ OR Date stopped: __ __/__ __/__ __ __ __
Month Day Year Month Day Year

1 ☐ Losartan

Dose: ________________ Frequency: ________ 1 ☐ Per day 2 ☐ Per week 3 ☐ Per month

Indication (reason): ____

Date started: __ __/__ __/__ __ __ __ OR Date stopped: __ __/__ __/__ __ __ __
Month Day Year Month Day Year

1 ☐ Valsartan

Dose: ________________ Frequency: ________ 1 ☐ Per day 2 ☐ Per week 3 ☐ Per month

Indication (reason): ____

Date started: __ __/__ __/__ __ __ __ OR Date stopped: __ __/__ __/__ __ __ __
Month Day Year Month Day Year

Diuretics (continued)

1 ☐ Eplerenone

Dose: ________________ Frequency: ________ 1 ☐ Per day 2 ☐ Per week 3 ☐ Per month

Indication (reason): ____

Date started: __ __/__ __/__ __ __ __ OR Date stopped: __ __/__ __/__ __ __ __
Month Day Year Month Day Year

Iron Replacement

1 ☐ Ferrous sulfate

Dose: ________________ Frequency: ________ 1 ☐ Per day 2 ☐ Per week 3 ☐ Per month

Indication (reason): ____

Date started: __ __/__ __/__ __ __ __ OR Date stopped: __ __/__ __/__ __ __ __
Month Day Year Month Day Year

Inflammatory Bowel Disease (IBD)

1 ☐ Mesalamine (DELZICOL, ROWASA®, Lialda®, PENTASA®, Canasa®, aspriso®, Asacol®)

Dose: ________________ Frequency: ________ 1 ☐ Per day 2 ☐ Per week 3 ☐ Per month

Indication (reason): ____

Date started: __ __/__ __/__ __ __ __ OR Date stopped: __ __/__ __/__ __ __ __
Month Day Year Month Day Year

1 ☐ Sulfasalazine (AZULFIDINE®)

Dose: ________________ Frequency: ________ 1 ☐ Per day 2 ☐ Per week 3 ☐ Per month

Indication (reason): ____

Date started: __ __/__ __/__ __ __ __ OR Date stopped: __ __/__ __/__ __ __ __
Month Day Year Month Day Year

1 ☐ Olsalazine (Dipentum®)

Dose: ________________ Frequency: ________ 1 ☐ Per day 2 ☐ Per week 3 ☐ Per month

Indication (reason): ____

Date started: __ __/__ __/__ __ __ __ OR Date stopped: __ __/__ __/__ __ __ __
Month Day Year Month Day Year

1 ☐ Balsalazide (COLASAL®, giazo®)

Dose: ________________ Frequency: ________ 1 ☐ Per day 2 ☐ Per week 3 ☐ Per month

Indication (reason): ____

Date started: __ __/__ __/__ __ __ __ OR Date stopped: __ __/__ __/__ __ __ __
Month Day Year Month Day Year

1 ☐ Alendronate (FOSAMAX®, Binosto®)

Dose: ________________ Frequency: ________ 1 ☐ Per day 2 ☐ Per week 3 ☐ Per month

Indication (reason): ____

Date started: __ __/__ __/__ __ __ __ OR Date stopped: __ __/__ __/__ __ __ __
Month Day Year Month Day Year

Osteoporosis (Bone Loss) (continued)

1 ☐ Clodronate (Bonesfos®)

Dose: ________________ Frequency: ________ 1 ☐ Per day 2 ☐ Per week 3 ☐ Per month

Indication (reason): ____

Date started: __ __/__ __/__ __ __ __ OR Date stopped: __ __/__ __/__ __ __ __
Month Day Year Month Day Year

1 ☐ Etidronate

Dose: ________________ Frequency: ________ 1 ☐ Per day 2 ☐ Per week 3 ☐ Per month

Indication (reason): ____

Date started: __ __/__ __/__ __ __ __ OR Date stopped: __ __/__ __/__ __ __ __
Month Day Year Month Day Year

1 ☐ Ibandronate (Boniva®)

Dose: ________________ Frequency: ________ 1 ☐ Per day 2 ☐ Per week 3 ☐ Per month

Indication (reason): ____

Date started: __ __/__ __/__ __ __ __ OR Date stopped: __ __/__ __/__ __ __ __
Month Day Year Month Day Year

1 ☐ Pamidronate

Dose: ________________ Frequency: ________ 1 ☐ Per day 2 ☐ Per week 3 ☐ Per month

Indication (reason): ____

Date started: __ __/__ __/__ __ __ __ OR Date stopped: __ __/__ __/__ __ __ __
Month Day Year Month Day Year

1 ☐ Risedronate (Actonel®, Atelvia®)

Dose: ________________ Frequency: ________ 1 ☐ Per day 2 ☐ Per week 3 ☐ Per month

Indication (reason): ____

Date started: __ __/__ __/__ __ __ __ OR Date stopped: __ __/__ __/__ __ __ __
Month Day Year Month Day Year

1 ☐ Zolendronic acid (Reclast®, ZOMETA®)

Dose: ________________ Frequency: ________ 1 ☐ Per day 2 ☐ Per week 3 ☐ Per month

Indication (reason): ____

Date started: __ __/__ __/__ __ __ __ OR Date stopped: __ __/__ __/__ __ __ __
Month Day Year Month Day Year

Steroids

1 ☐ Prednisone

Dose: ________________ Frequency: ________ 1 ☐ Per day 2 ☐ Per week 3 ☐ Per month

Indication (reason): ____

Date started: __ __/__ __/__ __ __ __ OR Date stopped: __ __/__ __/__ __ __ __
Month Day Year Month Day Year

Steroids (continued)

1 ☐ Budesonide

Dose: ________________ Frequency: ________ 1 ☐ Per day 2 ☐ Per week 3 ☐ Per month

Indication (reason): ____

Date started: __ __/__ __/__ __ __ __ (Month Day Year) OR Date stopped: __ __/__ __/__ __ __ __ (Month Day Year)

1 ☐ Hydrocortisone suppository (PROCTOCORT®, Hemril®, cortifoam®, Cortenema®, Colocort®)

Dose: ________________ Frequency: ________ 1 ☐ Per day 2 ☐ Per week 3 ☐ Per month

Indication (reason): ____

Date started: __ __/__ __/__ __ __ __ (Month Day Year) OR Date stopped: __ __/__ __/__ __ __ __ (Month Day Year)

Immunosuppression

1 ☐ Azathioprine (Imuran®)

Dose: ________________ Frequency: ________ 1 ☐ Per day 2 ☐ Per week 3 ☐ Per month

Indication (reason): ____

Date started: __ __/__ __/__ __ __ __ (Month Day Year) OR Date stopped: __ __/__ __/__ __ __ __ (Month Day Year)

1 ☐ Mercaptopurine

Dose: ________________ Frequency: ________ 1 ☐ Per day 2 ☐ Per week 3 ☐ Per month

Indication (reason): ____

Date started: __ __/__ __/__ __ __ __ (Month Day Year) OR Date stopped: __ __/__ __/__ __ __ __ (Month Day Year)

1 ☐ Methotrexate

Dose: ________________ Frequency: ________ 1 ☐ Per day 2 ☐ Per week 3 ☐ Per month

Indication (reason): ____

Date started: __ __/__ __/__ __ __ __ (Month Day Year) OR Date stopped: __ __/__ __/__ __ __ __ (Month Day Year)

1 ☐ CellCept (Mycophenolate)

Dose: ________________ Frequency: ________ 1 ☐ Per day 2 ☐ Per week 3 ☐ Per month

Indication (reason): ____

Date started: __ __/__ __/__ __ __ __ (Month Day Year) OR Date stopped: __ __/__ __/__ __ __ __ (Month Day Year)

1 ☐ Tacrolimus

Dose: ________________ Frequency: ________ 1 ☐ Per day 2 ☐ Per week 3 ☐ Per month

Indication (reason): ____

Date started: __ __/__ __/__ __ __ __ (Month Day Year) OR Date stopped: __ __/__ __/__ __ __ __ (Month Day Year)

Immunosuppression (continued)

1 ☐ Cyclosporine

Dose: ________________ Frequency: ________ 1 ☐ Per day 2 ☐ Per week 3 ☐ Per month

Indication (reason): ____

Date started: __ __/__ __/__ __ __ __ OR Date stopped: __ __/__ __/__ __ __ __
Month Day Year Month Day Year

Antibiotics

1 ☐ Ciprofloxacin

Dose: ________________ Frequency: ________ 1 ☐ Per day 2 ☐ Per week 3 ☐ Per month

Indication (reason): ____

Date started: __ __/__ __/__ __ __ __ OR Date stopped: __ __/__ __/__ __ __ __
Month Day Year Month Day Year

1 ☐ Metronidazole (Flagyl®)

Dose: ________________ Frequency: ________ 1 ☐ Per day 2 ☐ Per week 3 ☐ Per month

Indication (reason): ____

Date started: __ __/__ __/__ __ __ __ OR Date stopped: __ __/__ __/__ __ __ __
Month Day Year Month Day Year

1 ☐ Amoxicillin

Dose: ________________ Frequency: ________ 1 ☐ Per day 2 ☐ Per week 3 ☐ Per month

Indication (reason): ____

Date started: __ __/__ __/__ __ __ __ OR Date stopped: __ __/__ __/__ __ __ __
Month Day Year Month Day Year

1 ☐ Bactrim

Dose: ________________ Frequency: ________ 1 ☐ Per day 2 ☐ Per week 3 ☐ Per month

Indication (reason): ____

Date started: __ __/__ __/__ __ __ __ OR Date stopped: __ __/__ __/__ __ __ __
Month Day Year Month Day Year

1 ☐ Augmentin

Dose: ________________ Frequency: ________ 1 ☐ Per day 2 ☐ Per week 3 ☐ Per month

Indication (reason): ____

Date started: __ __/__ __/__ __ __ __ OR Date stopped: __ __/__ __/__ __ __ __
Month Day Year Month Day Year

Stomach Acid Inhibitors

1 ☐ Pantoprazole

Dose: ________________ Frequency: ________ 1 ☐ Per day 2 ☐ Per week 3 ☐ Per month

Indication (reason): ____

Date started: __ __/__ __/__ __ __ __ OR Date stopped: __ __/__ __/__ __ __ __
Month Day Year Month Day Year

Stomach Acid Inhibitors (continued)

1 ☐ Omeprazole

Dose: ________________ Frequency: ________ 1 ☐ Per day 2 ☐ Per week 3 ☐ Per month

Indication (reason): ______________________________

Date started: __ __/__ __/__ __ __ __ (Month Day Year) OR Date stopped: __ __/__ __/__ __ __ __ (Month Day Year)

Biological Agents

1 ☐ Adalimumab (HUMIRA®)

Dose: ________________ Frequency: ________ 1 ☐ Per day 2 ☐ Per week 3 ☐ Per month

Indication (reason): ______________________________

Date started: __ __/__ __/__ __ __ __ (Month Day Year) OR Date stopped: __ __/__ __/__ __ __ __ (Month Day Year)

1 ☐ Infliximab (Remicade®)

Dose: ________________ Frequency: ________ 1 ☐ Per day 2 ☐ Per week 3 ☐ Per month

Indication (reason): ______________________________

Date started: __ __/__ __/__ __ __ __ (Month Day Year) OR Date stopped: __ __/__ __/__ __ __ __ (Month Day Year)

1 ☐ Certolizumab (Cimzia®)

Dose: ________________ Frequency: ________ 1 ☐ Per day 2 ☐ Per week 3 ☐ Per month

Indication (reason): ______________________________

Date started: __ __/__ __/__ __ __ __ (Month Day Year) OR Date stopped: __ __/__ __/__ __ __ __ (Month Day Year)

1 ☐ Vedolizumab (Entyvio®)

Dose: ________________ Frequency: ________ 1 ☐ Per day 2 ☐ Per week 3 ☐ Per month

Indication (reason): ______________________________

Date started: __ __/__ __/__ __ __ __ (Month Day Year) OR Date stopped: __ __/__ __/__ __ __ __ (Month Day Year)

1 ☐ Natalizumab (TYSABRli®)

Dose: ________________ Frequency: ________ 1 ☐ Per day 2 ☐ Per week 3 ☐ Per month

Indication (reason): ______________________________

Date started: __ __/__ __/__ __ __ __ (Month Day Year) OR Date stopped: __ __/__ __/__ __ __ __ (Month Day Year)

1 ☐ Tofacitinib (Xelianz®)

Dose: ________________ Frequency: ________ 1 ☐ Per day 2 ☐ Per week 3 ☐ Per month

Indication (reason): ______________________________

Date started: __ __/__ __/__ __ __ __ (Month Day Year) OR Date stopped: __ __/__ __/__ __ __ __ (Month Day Year)

Biological Agents (continued)

1 ☐ Ustekinumab (Stelara®)

Dose: ________________ Frequency: ________ 1 ☐ Per day 2 ☐ Per week 3 ☐ Per month

Indication (reason): ____

Date started: __ __/__ __/__ __ __ __ (Month Day Year) OR Date stopped: __ __/__ __/__ __ __ __ (Month Day Year)

29. Indicate whether you have had any of the following surgeries. If you have not had this surgery, mark "No." If you have had the surgery, what was your approximate age at the time of the surgery and indicate if the surgery was cancer-related?

	Yes	No	Approximate age at time of surgery? Age at first surgery	Age at second surgery	Was the surgery cancer-related? Yes	No
**Appendix removal**	1 ☐	2 ☐	__ __	__ __	1 ☐	2 ☐
**Gallbladder removal**	1 ☐	2 ☐	__ __	__ __	1 ☐	2 ☐
**Liver resection (partial removal of the liver)**	1 ☐	2 ☐	__ __	__ __	1 ☐	2 ☐
**Bile duct surgery**	1 ☐	2 ☐	__ __	__ __	1 ☐	2 ☐
**Ulcer (stomach or intestinal)**	1 ☐	2 ☐	__ __	__ __	1 ☐	2 ☐
**Colon polyp removal**	1 ☐	2 ☐	__ __	__ __	1 ☐	2 ☐
**Colon**	1 ☐	2 ☐	__ __	__ __	1 ☐	2 ☐
**Uterine fibroid removal (women only)**	1 ☐	2 ☐	__ __	__ __	1 ☐	2 ☐
**Thyroid removal**	1 ☐	2 ☐	__ __	__ __	1 ☐	2 ☐
**Heart**	1 ☐	2 ☐	__ __	__ __	1 ☐	2 ☐
**Back**	1 ☐	2 ☐	__ __	__ __	1 ☐	2 ☐
**Hip/knee replacement**	1 ☐	2 ☐	__ __	__ __	1 ☐	2 ☐

Continues next page...

	Yes	No	Approximate age at time of surgery? Age at first surgery	Age at second surgery	Was the surgery cancer-related? Yes	No
**Other, specify:**______________	1 ☐	2 ☐	__ __	__ __	1 ☐	2 ☐
**Other, specify:**______________	1 ☐	2 ☐	__ __	__ __	1 ☐	2 ☐
**Other, specify:**______________	1 ☐	2 ☐	__ __	__ __	1 ☐	2 ☐
**Other, specify:**______________	1 ☐	2 ☐	__ __	__ __	1 ☐	2 ☐

30. Indicate whether you have had any of the following conditions. If you have not had this condition, mark "No." If you have had the condition, what was your approximate age at the time you were first diagnosed with the condition?

**Autoimmune Diseases**	Yes	No	Approximate age at first diagnoses?
Autoimmune hepatitis (AIH)	1 ☐	2 ☐	__ __
Autoimmune pancreatitis	1 ☐	2 ☐	__ __
Immunoglobulin G4-related cholangitis	1 ☐	2 ☐	__ __
Celiac disease	1 ☐	2 ☐	__ __
Pernicious anemia	1 ☐	2 ☐	__ __
Myasthenia gravis	1 ☐	2 ☐	__ __
Autoimmune hypothyroidism	1 ☐	2 ☐	__ __
Autoimmune hyperthyroidism	1 ☐	2 ☐	__ __
Type 1 diabetes mellitus	1 ☐	2 ☐	__ __
Addison's disease	1 ☐	2 ☐	__ __
Sjogren's syndrome	1 ☐	2 ☐	__ __
Systemic sclerosis	1 ☐	2 ☐	__ __

Continues next page...

Autoimmune Diseases (continued)	Yes	No	Approximate age at first diagnoses?
Scleroderma	1 ☐	2 ☐	__ __
Rheumatoid arthritis	1 ☐	2 ☐	__ __
Systemic lupus erythematosus	1 ☐	2 ☐	__ __
Psoriasis	1 ☐	2 ☐	__ __
Raynaud's phenomenon	1 ☐	2 ☐	__ __
Bullous pemphigoid	1 ☐	2 ☐	__ __
Vitiligo	1 ☐	2 ☐	__ __
Lichen planus	1 ☐	2 ☐	__ __
Polymyositis	1 ☐	2 ☐	__ __
Idiopathic thrombocytopenic purpura (ITP)	1 ☐	2 ☐	__ __
**Rheumatologic**			
Arthritis (osteoarthritis)	1 ☐	2 ☐	__ __
Fibromyalgia	1 ☐	2 ☐	__ __
**Gynecologic**			
Endometriosis	1 ☐	2 ☐	__ __
**Liver**			
Hepatitis A	1 ☐	2 ☐	__ __
Hepatitis B	1 ☐	2 ☐	__ __
Hepatitis C	1 ☐	2 ☐	__ __
Hemochromatosis (iron overload)	1 ☐	2 ☐	__ __
Alpha 1 antitrypsin deficiency (A1AT)	1 ☐	2 ☐	__ __
Drug induced liver injury (DILI)	1 ☐	2 ☐	__ __
Alcoholic liver disease	1 ☐	2 ☐	__ __

Continues next page...

Liver (continued)

	Yes	No	Approximate age at first diagnoses?
Nonalcoholic fatty liver disease (NAFLD)	1 ☐	2 ☐	__ __
Nonalcoholic steatohepatitis (NASH)	1 ☐	2 ☐	__ __
Other liver disease, specify: ______________	1 ☐	2 ☐	__ __
**Hematologic**			
Organ or bone marrow transplant	1 ☐	2 ☐	__ __
Bleeding disorder	1 ☐	2 ☐	__ __
Sickle cell anemia	1 ☐	2 ☐	__ __
**Infectious diseases**			
HIV (AIDS)	1 ☐	2 ☐	__ __
Tuberculosis	1 ☐	2 ☐	__ __
**Cancer**			
Thyroid	1 ☐	2 ☐	__ __
Lung	1 ☐	2 ☐	__ __
Breast	1 ☐	2 ☐	__ __
Esophageal	1 ☐	2 ☐	__ __
Pancreatic	1 ☐	2 ☐	__ __
Stomach	1 ☐	2 ☐	__ __
Colon or rectal	1 ☐	2 ☐	__ __
Liver	1 ☐	2 ☐	__ __
Bile duct	1 ☐	2 ☐	__ __
Women only: Uterine/endometrial	1 ☐	2 ☐	__ __
Women only: Cervical	1 ☐	2 ☐	__ __
Women only: Ovarian	1 ☐	2 ☐	__ __

Continues next page...

Cancer (continued)	Yes	No	Approximate age at first diagnoses?
Men only: Prostate	1 ☐	2 ☐	__ __
Men only: Testicular	1 ☐	2 ☐	__ __
Melanoma	1 ☐	2 ☐	__ __
Non-melanoma skin	1 ☐	2 ☐	__ __
Sarcoma	1 ☐	2 ☐	__ __
Bone	1 ☐	2 ☐	__ __
Leukemia	1 ☐	2 ☐	__ __
Lymphoma	1 ☐	2 ☐	__ __
Kidney	1 ☐	2 ☐	__ __
Urinary/bladder	1 ☐	2 ☐	__ __
Other, specify: ____________	1 ☐	2 ☐	__ __
**Neurologic**			
Alzheimer's disease	1 ☐	2 ☐	__ __
Parkinson's disease	1 ☐	2 ☐	__ __
Dementia	1 ☐	2 ☐	__ __
Migraine headaches	1 ☐	2 ☐	__ __
Stroke (CVA)	1 ☐	2 ☐	__ __
TIA (mini stroke)	1 ☐	2 ☐	__ __
Epilepsy (seizure disorder)	1 ☐	2 ☐	__ __
Narcolepsy	1 ☐	2 ☐	__ __
**Mental Health**			
Anxiety	1 ☐	2 ☐	__ __
Depression	1 ☐	2 ☐	__ __

Continues next page...

Mental Health (continued)

	Yes	No	Approximate age at first diagnoses?
Post-traumatic stress disorder (PTSD)	1 ☐	2 ☐	__ __
Biopolar disorder	1 ☐	2 ☐	__ __
Autism	1 ☐	2 ☐	__ __
Attention-deficit/hyperactivity disorder (ADD/ADHD)	1 ☐	2 ☐	__ __
Other psychiatric or mental illness, specify: ______________________	1 ☐	2 ☐	__ __

Eye

	Yes	No	Approximate age at first diagnoses?
Glaucoma	1 ☐	2 ☐	__ __
Cataracts	1 ☐	2 ☐	__ __
Abnormal distance vision	1 ☐	2 ☐	__ __
Lazy eye (amblyopia)	1 ☐	2 ☐	__ __
Misalignment, crossing, or wandering of the eyes (strabismus)	1 ☐	2 ☐	__ __
Macular degeneration	1 ☐	2 ☐	__ __

Cardiovascular

	Yes	No	Approximate age at first diagnoses?
Heart attack/myocardial infarction	1 ☐	2 ☐	__ __
Coronary artery disease	1 ☐	2 ☐	__ __
Peripheral arterial/vascular disease (PVD or PAD)	1 ☐	2 ☐	__ __
Congestive heart failure	1 ☐	2 ☐	__ __
Cardiomyopathy	1 ☐	2 ☐	__ __
Atrial fibrillation	1 ☐	2 ☐	__ __
Arrhythmia (ventricular tachycardia, premature atrial/ventricular arrhythmias, atrial flutter, etc.)	1 ☐	2 ☐	__ __

Continues next page...

Cardiovascular (continued)	Yes	No	Approximate age at first diagnoses?
Congenital heart disease	1 ☐	2 ☐	__ __
High blood pressure (hypertension)	1 ☐	2 ☐	__ __
High cholesterol (hyperlipidemia)	1 ☐	2 ☐	__ __
Blood clots in a vein	1 ☐	2 ☐	__ __
Blood clots in an artery	1 ☐	2 ☐	__ __
**Respiratory**			
Asthma	1 ☐	2 ☐	__ __
Chronic obstructive pulmonary disease (COPD)	1 ☐	2 ☐	__ __
Sleep apnea	1 ☐	2 ☐	__ __
Asbestosis	1 ☐	2 ☐	__ __
Pulmonary fibrosis	1 ☐	2 ☐	__ __
Immunoglobulin G4 (IgG4)-related disease of the lung	1 ☐	2 ☐	__ __
**Gastrointestinal**			
Acid reflux or gastroesophageal reflux disease (GERD)	1 ☐	2 ☐	__ __
Barrett's esophagus	1 ☐	2 ☐	__ __
Irritable bowel syndrome (IBS)	1 ☐	2 ☐	__ __
Lynch syndrome or HNPCC	1 ☐	2 ☐	__ __
Clostridium difficile infection (C. diff)	1 ☐	2 ☐	__ __
Fecal transplant procedure for treatment of C. diff	1 ☐	2 ☐	__ __
Other polyposis syndrome (FAP, Peutz-Jeghers, juvenile polyposis, etc.)	1 ☐	2 ☐	__ __

Continues next page...

Endocrine

	Yes	No	Approximate age at first diagnoses?
**Type 2 diabetes mellitus**	1 ☐	2 ☐	__ __

31. Do you have a family history of inflammatory bowel disease?

1 ☐ Yes 2 ☐ No

If you have a family history of inflammatory disease, which family members have been diagnosed? (Mark all that apply.)

1 ☐ Material (mother's side) 1 ☐ Children
1 ☐ Paternal (father's side) 1 ☐ Siblings

32. Have you been diagnosed with inflammatory bowel disease (IBD)?

1 ☐ Yes 2 ☐ No — **SKIP to question 39**

33. What type of IBD have you been diagnosed with?

1 ☐ Crohn's disease, please specify age at diagnosis: __ __ Age

2 ☐ Ulcerative colitis, please specify age at diagnosis: __ __ Age

3 ☐ Indeterminate colitis, please specify age at diagnosis: __ __ Age

34. Which of the following symptoms did you have at the time of your IBD diagnosis?

	Yes	No
**Abdominal pain**	1 ☐	2 ☐
**Diarrhea**	1 ☐	2 ☐
**Blood in stool**	1 ☐	2 ☐
**Fever/chills**	1 ☐	2 ☐

35. Have you ever been on chronic antibiotic therapy (2 weeks or more) for IBD?

1 ☐ Yes 2 ☐ No

If you have been on chronic antibiotic therapy (2 weeks or more), please list the name(s) of the drug(s), dose, frequency, and duration. (If more than once, please list the most recent.)

____

____

____

36. Have you been diagnosed with any of the following conditions?

	Yes	No	Approximate age at first diagnoses?
**Erythema nodosum (painful skin rash)**	1 ☐	2 ☐	__ __
**Pyoderma gangrenosum (ulcerating skin rash)**	1 ☐	2 ☐	__ __
**Aphthous stomatitis (ulcers of the mouth)**	1 ☐	2 ☐	__ __
**Episcleritis or schleritis (painful eye inflammation)**	1 ☐	2 ☐	__ __
**Uveitis (inflammation of the white of the eye)**	1 ☐	2 ☐	__ __
**Conjunctivitis**	1 ☐	2 ☐	__ __
**Ankylosing spondylitis (back pain and progressing back stiffness)**	1 ☐	2 ☐	__ __
**Venous thromboembolism (blood clots in veins)**	1 ☐	2 ☐	__ __
**Arterial thromboembolism (blood clots in arteries)**	1 ☐	2 ☐	__ __
**Kidney stones**	1 ☐	2 ☐	__ __
**Osteopenia (weakening of the bones)**	1 ☐	2 ☐	__ __

37. Have you had complications from inflammatory bowel disease?

1 ☐ Yes 2 ☐ No

What complications have you had from inflammatory bowel disease? If you had this complication, what was your approximate age(s) at the time you experienced this complication? (Mark all that apply.)

	Age 1	Age 2	Age 3
1 ☐ Toxic megacolon (enlargement of the colon)	__ __	__ __	__ __
1 ☐ Perforation of the colon	__ __	__ __	__ __
1 ☐ Cancer of the colon or rectum	__ __	__ __	__ __

38. Have you ever had surgery for inflammatory bowel disease?

1 ☐ Yes 2 ☐ No

Did you have part or all of your colon removed?

1 ☐ Yes 2 ☐ No

What was your approximate age when you had part or all of your colon removed?

__ __ Age

Did you have part of your small intestine removed?

1 ☐ Yes 2 ☐ No

What was your approximate age when you had part of your small intestine removed?

__ __ Age

Have you had surgery for perianal (area around the anus complications?

1 ☐ Yes 2 ☐ No

What was your approximate age when you had surgery for perianal (area around the anus) complications?

__ __ Age

Do you have a permanent ileostomy?

1 ☐ Yes 2 ☐ No

What was your approximate age when you had a permanent ileostomy?

__ __ Age

Do you have a pouch (ileal pouch anal anastomosis)?

1 ☐ Yes 2 ☐ No

What was your approximate age when you got a pouch (ileal pouch anal anastomosis)?

__ __ Age

39. Did/do you have repeated urinary tract infections?

1 ☐ Yes 2 ☐ No

How often did/do you have repeated urinary tract infections?

1 ☐ Once every 2 years 2 ☐ Once every year 3 ☐ 2 or more times a year

40. Are you a recipient of a liver transplant?

1 ☐ Yes 2 ☐ No

Year(s) of transplant. (Please enter a 4-digit year, for example, 2020.)

_ _ _ _ Year _ _ _ _ Year _ _ _ _ Year _ _ _ _ Year _ _ _ _ Year

41. Are you a transplant recipient of organs or tissues other than liver?

1 ☐ Yes 2 ☐ No

What kind(s) of transplants did you have and in what year(s).
(If more than once, list all the times you have undergone a transplant other than liver. Please enter a 4-digit year, for example, 2020.)

Type of transplant: ______________ ______________ ______________

Year of transplant: _ _ _ _ Year _ _ _ _ Year _ _ _ _ Year

42. Have you ever been treated with chemotherapy for cancer?

1 ☐ Yes 2 ☐ No

If yes, please indicate the most recent date of treatment.
(If you don't know the exact date, please enter what you do know. If you do not recall the date, please mark "I do not recall the date.")

__ __/__ __/__ __ __ __ 1 ☐ I do not recall the date
Month Day Year

43. Have you ever been treated with radiation for any condition, including cancer?

1 ☐ Yes 2 ☐ No

If yes, please indicate the most recent date of treatment.
(If you don't know the exact date, please enter what you do know. If you do not recall the date, please mark "I do not recall the date.")

__ __/__ __/__ __ __ __ 1 ☐ I do not recall the date
Month Day Year

44. **Were you born by cesarean section (c-section) or vaginal birth?**

1 ☐ Cesarean section 2 ☐ Vaginal 3 ☐ Don't know

45. **Did you take antibiotics before the age of one?**

1 ☐ Yes 2 ☐ No 3 ☐ Don't know

46. **As an infant, were you bottle fed, breast fed, or both?**

1 ☐ Bottle fed 2 ☐ Breast fed 3 ☐ Both 4 ☐ Don't know

47. **Do you have allergies?**

1 ☐ Yes 2 ☐ No

What kind of allergies do you have? (Mark all that apply.)

1 ☐ Food allergies such as shellfish or nuts
1 ☐ Grasses, pollen, or dust
1 ☐ Pets
1 ☐ Insect strings or bites
1 ☐ Medication
1 ☐ Other, please specify: ______________________

48. **Are you currently or have you ever participated in a clinical trial either at Mayo Clinic or elsewhere?**

1 ☐ Yes 2 ☐ No — **Skip to question 49**

CLINICAL TRIAL #1:

Please provide:

Name of trial: ______________________

Name of investigator: ______________________

Approximate date(s) of participation: ______________________

Did this clinical trial evaluate a drug or device?

1 ☐ Drug 2 ☐ Device 1 ☐ No

What was the name of the device? ______________________

Do you know if you received the active drug or the placebo?

1 ☐ Received the active drug 2 ☐ Received the placebo 3 ☐ Don't know

What was the name of the drug? ______________________

Continues next page...

CLINICAL TRIAL #2:

Please provide:

Name of trial: ______________________________

Name of investigator: ______________________________

Approximate date(s) of participation: ______________________________

Did this clinical trial evaluate a drug or device?

1 ☐ Drug 2 ☐ Device 1 ☐ No

What was the name of the device? ______________________________

Do you know if you received the active drug or the placebo?

1 ☐ Received the active drug 2 ☐ Received the placebo 3 ☐ Don't know

What was the name of the drug? ______________________________

CLINICAL TRIAL #3:

Please provide:

Name of trial: ______________________________

Name of investigator: ______________________________

Approximate date(s) of participation: ______________________________

Did this clinical trial evaluate a drug or device?

1 ☐ Drug 2 ☐ Device 1 ☐ No

What was the name of the device? ______________________________

Do you know if you received the active drug or the placebo?

1 ☐ Received the active drug 2 ☐ Received the placebo 3 ☐ Don't know

What was the name of the drug? ______________________________

FEMALES ONLY SECTION (MALES — Skip to question 54)

49. Have you had your uterus removed or was your last menstrual period more than 12 months ago?

1 ☐ Yes 2 ☐ No

How old were you when you entered menopause? __ __ Age

What was the reason your periods stopped? (Mark ONLY one.)

1 ☐ Natural menopause (change of life)
2 ☐ Because of hysterectomy or removal of ovaries (or both)
3 ☐ Took medication that stopped my period
4 ☐ Radiation/chemotherapy
5 ☐ Other, please specify: ______________________________

50. Have you ever been pregnant?

1 ☐ Yes 2 ☐ No

How many times have you been pregnant? (Include all stillbirths, miscarriages, ectopic, or tubal pregnancies, induced abortions, and current pregnancy, if applicable.)

☐ 1 ☐ 3 ☐ 5 ☐ 7 ☐ 9 or more
☐ 2 ☐ 4 ☐ 6 ☐ 8

How many pregnancies resulted in a live birth? (Count multiples as one birth.)

☐ 0 **— Skip to question 51**
☐ 1 ☐ 3 ☐ 5 ☐ 7 ☐ 9 or more
☐ 2 ☐ 4 ☐ 6 ☐ 8

What was your age when your first child was born? __ __ Age

How many children did you breastfeed for more than 1 month? __ __ Number of children

What was your age when your last child was born? __ __ Age

Are you pregnant right now? (Mark all that apply.)

1 ☐ Yes 2 ☐ No

What is your expected due date? (If you do not know the exact date, please enter what you do know.)

__ __/__ __/__ __ __ __
Month Day Year

51. Have you ever used birth control pills, patches, implants, or shots?

1 ☐ Yes, currently 2 ☐ Yes, but not currently 3 ☐ No

What is the total time you used birth control pills, patches, implants, or shots?

1 ☐ 6 months or less
2 ☐ 7 to 11 months
3 ☐ 1 to 2 years
4 ☐ 3 to 5 years
5 ☐ 6 to 10 years
6 ☐ 11 years or more

52. Have you ever taken hormone replacement therapy other than birth control pills, (e.g., estrogen, estrogen/progesterone combination)?

1 ☐ Yes, currently 2 ☐ Yes, but not currently 3 ☐ No

What type are you taking now or have you taken most recently?
(Mark all that apply.)

1 ☐ Estrogen alone
2 ☐ Estrogen and progesterone combination (e.g., Provera or Prempo)
3 ☐ Other, please specify: ______________________________
4 ☐ Don't know

How old were you when you first began taking any hormone therapy?

__ __ Age

How many years have you taken hormone therapy?

__ __ Years

53. **Have you ever taken tamoxifen (Nolvadex)?**

1 ☐ Yes, currently 2 ☐ Yes, but not currently 3 ☐ No 4 ☐ Don't know

What type are you taking now or have you taken most recently?
(Mark all that apply.)

1 ☐ Less than 1 month
2 ☐ 1 to 6 months
3 ☐ 7 to 11 months
4 ☐ 1 to 2 years
5 ☐ 3 to 4 years
6 ☐ 5 years or more
7 ☐ Don't know how long

MALES ONLY SECTION

54. **Have you ever had a prostate-specific antigen (PSA) blood test?**

1 ☐ Yes 2 ☐ No 3 ☐ Don't know

Did you ever have an abnormal test?

1 ☐ Yes 2 ☐ No 3 ☐ Don't know

When was the last time you had an abnormal test?

1 ☐ A year ago or less
2 ☐ More than 1, but not more than 2 years ago
3 ☐ More than 2, but not more than 5 years ago
4 ☐ More than 5 years ago
5 ☐ Don't know

Thank you for completing this survey!

Please return your completed survey in the envelope provided.

If your envelope is missing, please mail your survey to:

Survey Research Center
Harwick 7
200 First Street SW
Rochester MN 55905

©2021 Mayo Foundation for Medical Education and Research. All rights reserved. MAYO, MAYO CLINIC and the triple-shield Mayo logo are trademarks and service marks of MFMER.

MC4269-000

(THIS PAGE IS FOR SURVEY RESEARCH TRACKING AND FILING PURPOSES ONLY)

LIVER BIOBANK QUESTIONNAIRE

Investigator: Erik Schlicht, CCRP

Version as of:
January 13, 2021
January 14, 2021 LJH

Coding Check:

Include:
